# Evaluation of Reference Genes for RT qPCR Analyses of Structure-Specific and Hormone Regulated Gene Expression in *Physcomitrella patens* Gametophytes

**DOI:** 10.1371/journal.pone.0070998

**Published:** 2013-08-09

**Authors:** Aude Le Bail, Sebastian Scholz, Benedikt Kost

**Affiliations:** Cell Biology & Erlangen Center of Plant Science (ECROPS), Friedrich Alexander University, Erlangen, Germany; University of Nottingham, United Kingdom

## Abstract

The use of the moss *Physcomitrella patens* as a model system to study plant development and physiology is rapidly expanding. The strategic position of *P. patens* within the green lineage between algae and vascular plants, the high efficiency with which transgenes are incorporated by homologous recombination, advantages associated with the haploid gametophyte representing the dominant phase of the *P. patens* life cycle, the simple structure of protonemata, leafy shoots and rhizoids that constitute the haploid gametophyte, as well as a readily accessible high-quality genome sequence make this moss a very attractive experimental system. The investigation of the genetic and hormonal control of *P. patens* development heavily depends on the analysis of gene expression patterns by real time quantitative PCR (RT qPCR). This technique requires well characterized sets of reference genes, which display minimal expression level variations under all analyzed conditions, for data normalization. Sets of suitable reference genes have been described for most widely used model systems including e.g. *Arabidopsis thaliana*, but not for *P. patens*. Here, we present a RT qPCR based comparison of transcript levels of 12 selected candidate reference genes in a range of gametophytic *P. patens* structures at different developmental stages, and in *P. patens* protonemata treated with hormones or hormone transport inhibitors. Analysis of these RT qPCR data using GeNorm and NormFinder software resulted in the identification of sets of *P. patens* reference genes suitable for gene expression analysis under all tested conditions, and suggested that the two best reference genes are sufficient for effective data normalization under each of these conditions.

## Introduction

Together with all other Bryophytes, mosses form an evolutionary important group of multi-cellular, non-vascular land plants, which link green algae to vascular plants [1]. Although *Physcomitrella patens* has been studied by pioneering researchers for more than 80 years, only during the last decade the enormous potential of this moss as an experimental system for the investigation of plant development and physiology has begun to be fully recognized and exploited. *P. patens* is the only currently available plant model system that allows effective gene targeting (e.g. gene knock-out or GFP knock-in experiments) based on efficient genomic integration of transgenes by homologous recombination [2], and therefore provides unique opportunities for the investigation of plant gene functions. The dominant phase of the *P. patens* life cycle is the haploid gametophyte, which substantially facilities the analysis of mutant phenotypes induced by gene knock-out, or by the application of other forward or reverse genetic strategies. The attractiveness of *P. patens* as an experimental model is further enhanced by the availability of a fully assembled and annotated genome sequence [3].

The haploid gametophyte corresponds to the photosynthetically active green moss plant and represents the dominant phase of the *P. patens* life cycle. It is composed of filamentous protonemata and gametophores, which are leafy shoots with attached rhizoids. Gametophyte development starts with spore germination that leads to the outgrowth of protonemal filaments, single-cell files that elongate by tip-growth and serial transverse divisions of the apical cell [4]. Division of sub-apical protonemal cells can also occur and results either in the establishment of lateral protonemal branches, or in the formation of a bud that further develops into a leafy shoot. From the epidermis at the basal end of developing leafy shoots, root-like structures called rhizoids eventually grow out and into the substrate. Like protonemal filaments, rhizoids are single-cell files that elongate based on the tip growth and division of apical cells.

Plant hormones play crucial roles in *P. patens* development [5], [6], which continue to be under intense investigation. As protonemal filaments mature, the cells of which they are composed undergo a transition from chloronemal to caulonemal phenotype. Auxin mediates this transition and appears to be required for the development of cells displaying caulonemal characteristics [7], [8]. Bud formation and subsequent gametophore development is induced by cytokinins at low concentration in young chloronemal protonemata and at higher concentrations also in more mature caulonemal protonemata [9]. Treatment with abscisic acid (ABA) enhances the desiccation tolerance of *P. patens* protonemata [10], presumably in part because this hormone triggers the formation of brachycytes and tmema cells [11], sort of vegetative spores that are highly resistant to unfavorable conditions.

Expression pattern analysis of specific genes is an essential approach to enhance our understanding of the genetic control and the hormonal regulation of the development of the different structures constituting the *P. patens* gametophyte. Real-time quantitative PCR (RT qPCR) is currently the most widely employed method to determine expression patterns of individual genes independently of the experimental system used [12]–[14], as this technique is highly sensitive and allows the relatively rapid generation of reproducible, quantitative data sets [15], [16]. Because of these advantages, RT qPCR is also the method of choice for the confirmation of global gene expression analysis based on microarray technology [14], [17], [18].

RT qPCR analysis of regulated target gene expression relies on data normalization based on reference genes, whose expression level ideally is not changing under the different conditions that modulate target gene activity. Because ideal references genes generally can’t be identified, it is common practice to normalize RT qPCR data based on the use of multiple reference genes that have been specifically selected for high expression stability under all analyzed conditions. A number of software solutions, such as GeNorm [19], NormFinder [20] and BestKeeper [21], are available to aid the selection of suitable reference genes from pools of tested candidate genes. Genes coding for proteins with presumed housekeeping functions are preferentially included into candidate gene pools, because the activity of such genes is expected to be relatively stable under variable conditions. Extensive efforts have resulted in the identification of sets of reference genes suitable for RT qPCR analysis of gene expression under different conditions in a large variety of mammalian and other animal systems, including for example human [22], [23] cow [24], pig [25] and fish [26]. Sets of suitable reference genes have also been described in the literature for many of the most important plant model systems, such as *Arabidopsis thaliana* [27], rice [28]–[30], poplar [31] and tobacco [32], for less intensly studied plants such as peach [33], *Salvia miltiorrhiza* [34], *Bupleurum chinense* [35], and *Brachypodium distachyon* [36], and even for the brown alga *Ectocarpus siliculosus* [37].

Surprisingly, systematic efforts to identify reference genes suitable for RT qPCR analysis of developmentally and/or hormonally regulated gene expression in *P. patens* have not been reported to date. To remedy this situation, we have employed RT qPCR to determine the expression levels of 12 selected candidate reference genes in a range of gametophytic *P. patens* structures at different developmental stages (42 day old whole gametophytes, leafy shoots and rhizoids; 7 day old protonemata) as well as in *P. patens* protonemata treated with hormones (auxins, a cytokinin, ABA) or with auxin transport inhibitors. RT qPCR data obtained were analyzed using GeNorm and NormFinder software to identify sets of *P. patens* reference genes suitable for gene expression analysis under the different tested conditions, and to determine the number of reference genes required for effective data normalization under each of these conditions.

## Results

### RNA Sampling

To compare expression levels of selected candidate reference genes in a range of gametophytic *P. patens* structures at different developmental stages (figure S1), and in *P. patens* protonemata treated with hormones or hormone transport inhibitors (figure S2), total RNA was isolated from various tissues. Separate RNA samples were prepared from 7 day old protonemata grown on cellophane either on plain BCDA medium, or in the presence of auxins (IAA [indole-3-acetic acid]; NAA [1-naphthaleneacetic acid]), auxin transport inhibitors (NPA [1-N-naphthylphthalamic acid]; TIBA [2,3,5-triiodobenzoic acid]), a cytokinin (6-BAP [6-benzylaminopurine]) or ABA at concentrations, at which these hormones or transport inhibitors affect the development of moss [7]–[11] (figure S2) and/or vascular plants [38]. Treatment of *P. patens* with NPA was reported to interfere with auxin transport and morphogenesis in diploid sporophytes, but did not affect leafy shoots, which do not seems to display polar auxin transport [39]. No information is available in the literature about effects of auxin transport inhibitors on other *P. patens* structures. RNA was also purified from 42 day old whole gametophytes grown on BCD medium, which are composed of gametophores (leafy shoots with attached rhizoids) and protonemata, as well as from leafy shoots and from rhizoids isolated from such gametophytes (figure S1). Table 1 provides an overview of the 10 different RNA samples collected for candidate reference gene expression analysis. To be able to correct for biological variation, each RNA sample was prepared three times under identical conditions.

**Table 1:** RNA samples analyzed by RT qPCR.

N°	Structures	Hormones or hormone transport inhibitors	Age of culture	Medium
**1**	protonemata	–	7 days	BCDA
**2**	protonemata	5 µM Indole-3-acetic acid	7 days	BCDA
**3**	protonemata	1 µM 1-Naphthaleneacetic acid	7 days	BCDA
**4**	protonemata	5 µM N-1-Naphthylphthalamic acid	7 days	BCDA
**5**	protonemata	5 µM 2,3,5-Triiodobenzoic acid	7 days	BCDA
**6**	protonemata	5 µM Abscisic acid	7 days	BCDA
**7**	protonemata	5 µM 6-Benzylaminopurine (Cytokinin)	7 days	BCDA
**8**	rhizoids	–	42 days	BCD
**9**	leafy shoots	–	42 days	BCD
**10**	whole plants	–	42 days	BCD

Plant material was grown under continuous light at 25°C either on BCDA covered with cellophane (protonemal cultures) or on BCD (rhizoids, leafy shoots, whole plants).

### Expression Range of Candidate Reference Genes

**Figure 1:**
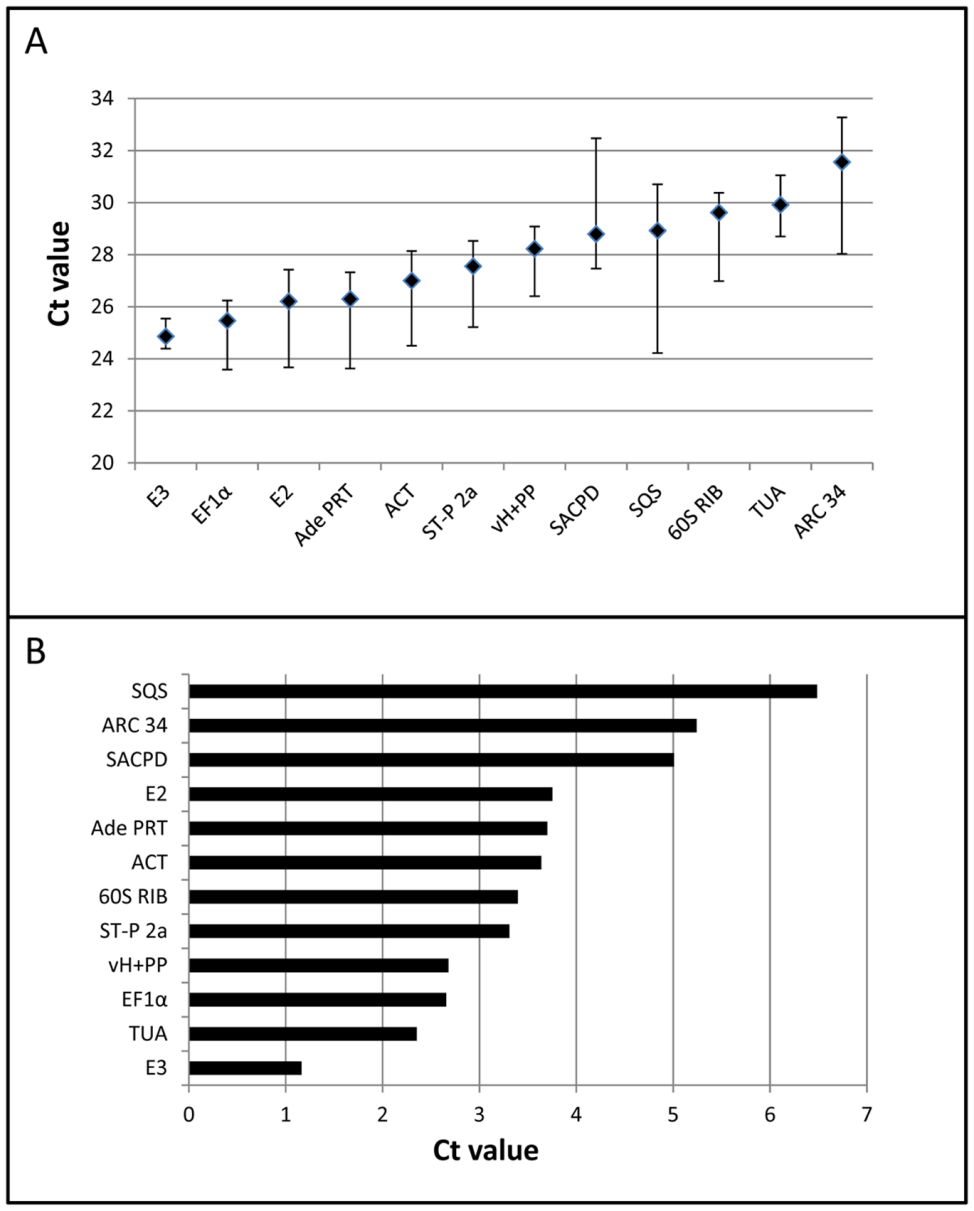
Expression range of *P. patens* candidate reference genes as determined by RT qPCR. (A) Mean Ct value (diamonds) as well as range between minimal and maximal Ct value representing expression levels of each candidate reference gene (table 2) in a number of gametophytic *P. patens* structures at different developmental stages, and in *P. patens* protonemata treated with hormones or hormone transport inhibitors (table 1). Candidate genes are arranged such that mean Ct values are increasing from left to right. (B) Ranking of candidate reference genes according to the difference between minimal and maximal Ct value.

RT qPCR analysis of all RNA samples (table 1) was performed to determine the expression levels of the closest *P. patens* homologs (table 2) of 12 genes described in literature as suitable algal or plant reference genes [37], [40]–[46]. The proteins encoded by most of these genes presumably have very different housekeeping functions as components or regulators of the cytoskeleton (actin 5 [ACT], a tubulin 1 [TUA], arp2/3 subunit 34 [ARC34]), or in central cellular processes such as translation (elongation factor 1α [EF1α], 60 s ribosomal protein [60S RIB]), protein degradation (ubiquitin-conjugation enzyme E2 [E2], E3 ubiquitin ligase [E3]), metabolism (squalene synthase [SQS], stearoyl-acyl-carrier protein desaturase [SCPA]) or signaling (serine threonine protein phosphatase 2a regulatory subunit [ST-P 2a], adenine phosphoribosyltransferase [Ade PRT], v-Type h_+_-translocating pyrophosphatase [vH+PP]). A number of the selected genes, including ACT, EF1α and TUA, are among the most commonly used reference genes for RT qPCR analysis in higher plants [47]–[49] and in *P. patens* [50]–[52]. Mean Ct value as well as the difference between minimal and maximal Ct value for the amplification of fragments of each *P. patens* candidate reference gene (table 2) from the 10 different RNA samples (table 1) are shown in figure 1. Mean Ct values covered a wide range from 24.5 (E3) to 31.5 (ARC34) corresponding to a difference a gene expression level of 128 times (2^31.5–24.5^; figure 1a). According to our experience, the Ct values for most characterized *P. patens* genes, apart from those displaying highest and lowest expression levels, fall within this range. The difference between minimal and maximal Ct value for individual reference genes ranged from 1.2 (2x difference in expression level, E3) to 6.5 (90x difference in expression level, SQS) (figure 1b), did not obviously correlate with mean gene expression level (figure 1a) and was relatively small for the commonly used plant and *P. patens* reference genes TUA, EF1α and ACT.

**Table 2:** *P. patens* candidate reference genes.

Gene Symbol	Gene description	Accession number	Oligonucleotides forward-reverse	PCR efficiency (%)	Regression coefficient (*R^2^*)	Amplicon T_m_ (°C)	Amplicon size (bp)
**60S RIB**	60s ribosomal protein	Phypa_438496	ACGGACATTGCATTTAAGACCT GTCGATTACCTGTGGAGAAGAC	97.1	0.987	82.0	210
**ACT**	actin 5	Phypa_459310	ACCGAGTCCAACATTCTACC GTCCACATTAGATTCTCGCA	97.7	0.998	82.0	112
**Ade PRT**	adenine phosphoribosyltransferase	Phypa_443007	AGTATAGTCTAGAGTATGGTACCG TAGCAATTTGATGGCAGCTC	99.31	1.000	83.0	130
**ARC34**	arp2/3 actin-organizing complexsubunit arc34	Phypa_453960	ACAAAGATAGCTTCCTTGCG AACAAGATTATCAGCATCTGGAG	101.1	0.997	82.25	104
**E2**	ubiquitin-conjugating enzyme E2	Phypa_430768	TACGGACCCTAATCCAGATGAC CAACCCATTGCATACTTCTGAG	94.4	0.994	81.8	116
**E3**	E3 ubiquitin ligase	Phypa_424619	TGAACTGATGGGACTAGAGG TCTTTGCTTACTCACGATGAC	96.8	0.998	81.0	161
**EF1α**	elongation factor 1α	Phypa_439314	AATCATACATTTCACCTCGCC GATCAGTGGGTAGAAGTGAC	95.9	0.994	83.0	111
**SACPD**	stearoyl-acyl-carrier proteindesaturase	Phypa_447144	CATCTGTAATGTGCATAGTGGG TATGTACCATTCACAGTATACCCT	89.2	0.995	80.0	159
**SQS**	squalene synthase	Phypa_428118	AGGTTTACACTGTCTGAACGA CAGAATCGAAGATTTGGTTGGT	103.4	0.997	80.5	104
**ST-P 2a**	serine threonine proteinphosphatase 2a regulatory subunit	Phypa_451689	GTCTAGTTAGTCCTTTGGTCCT GCCTATTTCTATAATGACTCCGT	104.5	0.994	82.0	124
**TUA**	alpha Tubulin 1	Phypa_437321	CTGTTACTTTATGTTCGGAGC ACACTAGTAAGTAAACGCGG	94.8	0.997	81.5	163
**vH+PP**	v-Type h(+)-translocating pyrophosphatase	Phypa_178090	GCAAGCCAATCAGTACACTC ATCTTAGCCAACAACCAATAACC	93.86	0.990	83.0	161

Accession numbers according to www.cosmoss.org are indicated. For all amplicons, PCR efficiency and R^2^ values were measured and found to be within the range required for reliable RT qPCR (90–110% and 0.99–1.00, respectively). Key amplicon characteristics (melting temperature T_m_, size in base pairs) are indicated on the right.

### GeNorm Analysis of Candidate Reference Gene Expression Stability

**Figure 2:**
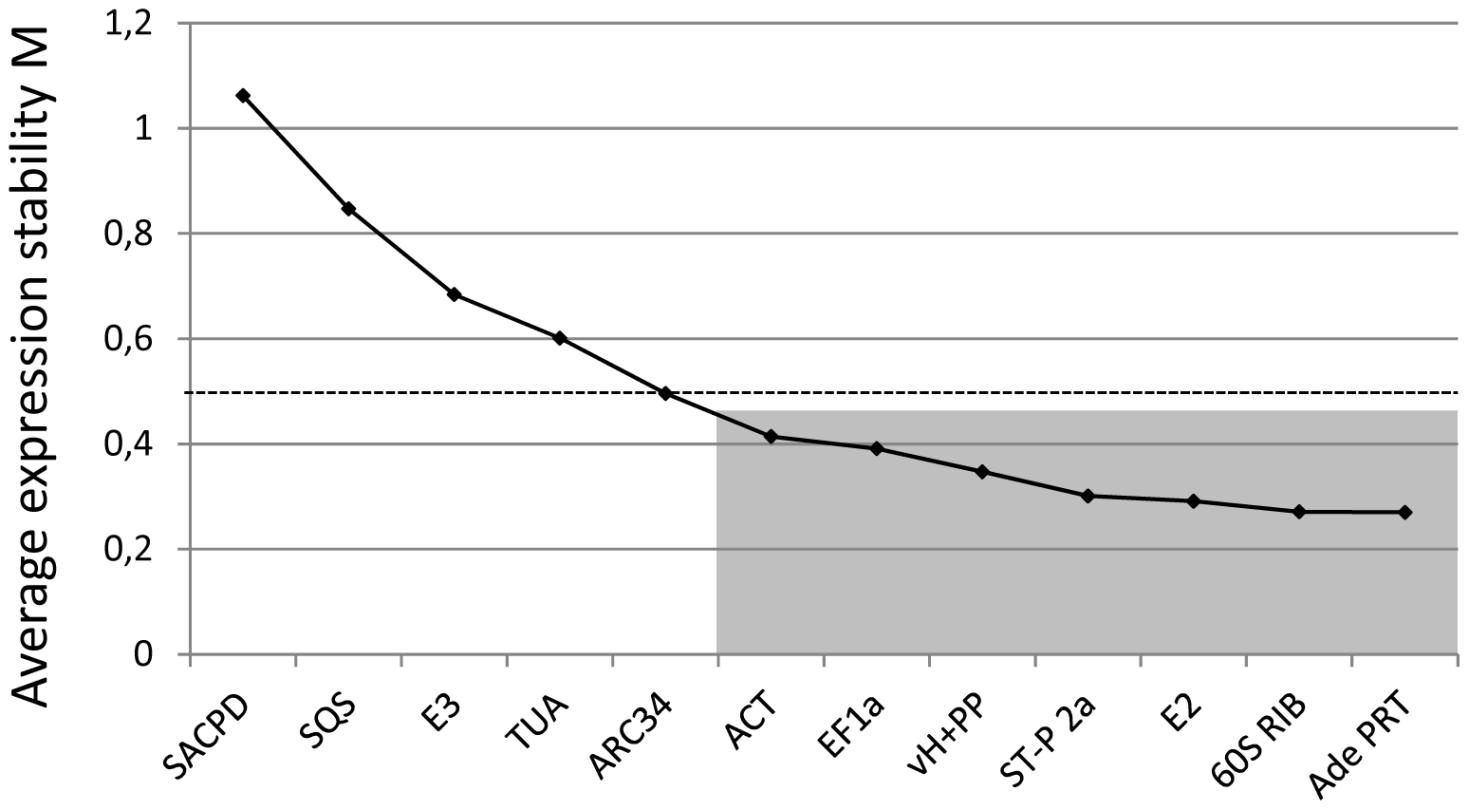
Global ranking of candidate reference gene expression stability based on GeNorm analysis. *M* values displayed were computed by GeNorm [19] based on data obtained by RT qPCR analysis of candidate reference gene expression in a range of gametophytic *P. patens* structures at different developmental stages, and in *P. patens* protonemata treated with hormones or hormone transport inhibitors (figure 1). Low *M* values indicate stable gene expression. Reference genes assigned *M* values below 0.5 (dotted line) are considered stably expressed and suitable for RT qPCR data normalization. The grey area marks the 7 best reference genes.

GeNorm software is widely employed to identify suitable reference genes from pools of candidate genes based on the principle that the expression ratio of ideal reference genes is expected to be identical in all analyzed samples [19]. The application of this principle relies on the use of independently regulated reference genes, as co-regulation obviously would result in an overestimation of expression stability. For each tested reference gene, GeNorm software computes an average expression stability value (*M*), which is a function of the average pairwise variation in the expression level of an individual gene as compared to all other reference genes. Stepwise exclusion of the reference gene with the highest *M* value (lowest expression stability) and computation of new *M* values for the remaining genes results in a ranking of the expression stability of all analyzed candidate reference genes. Published GeNorm algorithms do not allow a distinction between the two most suitable reference genes with the lowest *M* values [19]. However, the latest version of GeNorm (GeNorm^PLUS^) also ranks the two best reference genes based on additional unpublished algorithms and data collected during the stepwise exclusion procedure (Biogazelle, Zwijnaarde, Belgium; personal communication). Figure 2 presents the output generated by this version of GeNorm based on data obtained by the RT qPCR analysis of *P. patens* candidate reference gene expression described above (figure 1). To our knowledge, none of these reference genes are co-regulated. Ade PRT and 60S RIB were identified as the most stably expressed reference genes with the lowest, almost identical *M* values. Of the 12 tested reference genes, 8 were assigned *M* values below 0.5 and can therefore be considered stably expressed, suitable reference genes. Interestingly, all three commonly used plant reference genes were not ranked very highly. ACT and EF1α displayed the 2^nd^ (0.414) and 3^rd^ (0.391) highest *M* value, respectively, among the group of suitable reference genes, whereas TUA with an *M* value of 0.601 was not even included into this group.

**Figure 3:**
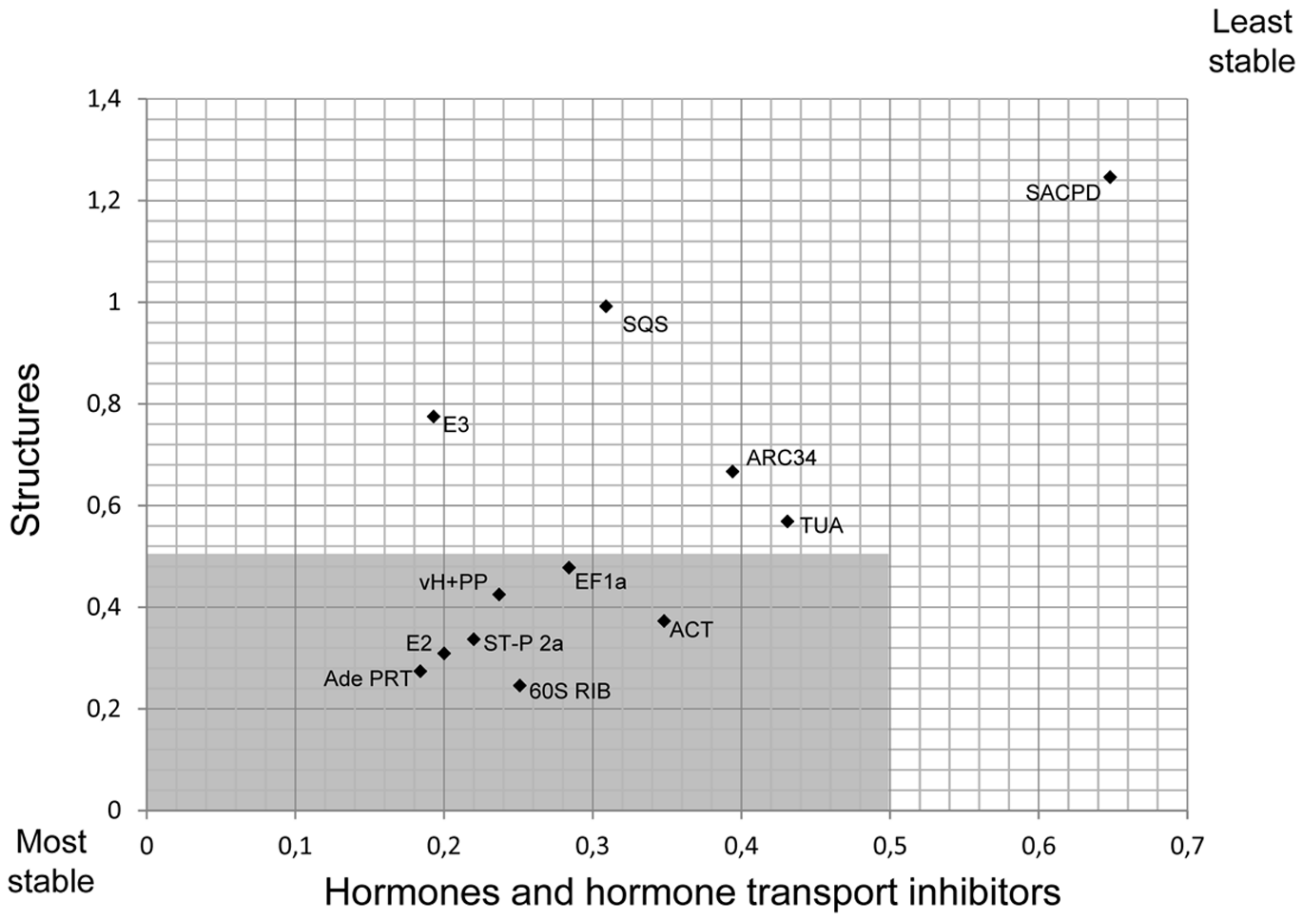
Separate GeNorm ranking of candidate reference gene expression stability in different gametophytic *P. patens* structures and in *P. patens* protonemata treated with hormones or hormone transport inhibitors. Plot showing *M* values separately computed by GeNorm for the two different groups of RNA samples (1, 8–10 and 1–7, table 1) based on the same RT qPCR data as analyzed in figures 1 and 2. Seven reference genes (grey area) were assigned *M* values below 0.5 independently of whether gene expression was analyzed in different structures, or in protonemata treated with hormones or hormone transport inhibitors. These genes are considered suitable for the RT qPCR data normalization under both conditions.

GeNorm was also employed to separately evaluate the stability of candidate reference gene expression only in different *P. patens* structures (RNA samples 1, 8–10; table 1) or in *P. patens* protonemata treated with hormones or hormone transport inhibitors (RNA samples 1–7; table 1). The results of this analysis are shown in figure 3. The expression of all candidate reference genes with the exception of 60S RIB was clearly more variable in different structures, than in protonemata treated with hormones or hormone transport inhibitors. The two reference genes found to be most stably expressed in different structures were again Ade PRT and 60S RIB. However, as 60S RIB expression displayed a relatively high variability in protonemata treated with hormones or hormone transport inhibitors, the two reference genes with the lowest *M* values in these protonemata were Ade PRT and E3. A group of 7 genes were classified as stably expressed suitable reference genes with *M* values below 0.5 independently of whether different structures or protonemata treated with hormones or hormone transport inhibitors were analyzed (figure 3, grey area). The same 7 genes were also identified as the most suitable reference genes when all 10 RNA samples were collectively analyzed (figure 2, grey area). However, under these conditions an additional gene (ARC34) was assigned an *M* value just below threshold (0.496; figure 2).

### GeNorm Estimation of the Number of Reference Genes Required for Normalization

**Figure 4:**
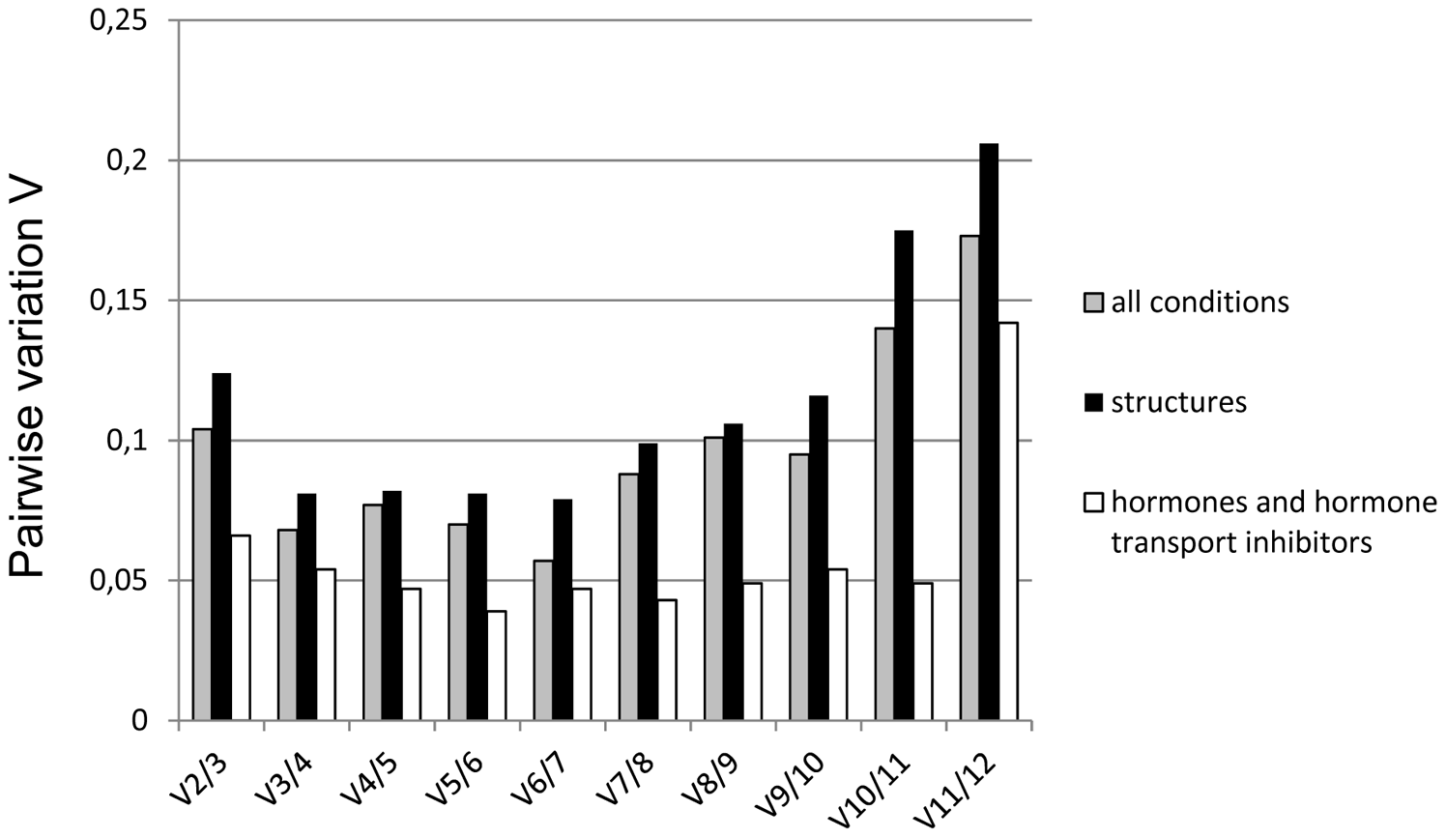
GeNorm estimation of the number of reference genes required for normalization. A comparison is shown of the normalization factor (*NF*) computed by GeNorm based on geometric averaging of the expression levels of the two best reference genes with additional *NF*s obtained after the stepwise inclusion into the analysis of more reference genes in the order of their *M* value ranking. Pairwise variation (*Vn*/*n+1*) between *NF*s computed based on *n* and *n+1* reference genes is plotted. *V2/3* values below 0.15 were obtained when RT qPCR data for reference gene expression in different structures, or in protonemata treated with hormones or hormone transport inhibitors, were analyzed separately (black columns: structures, white columns: hormones and hormone transport inhibitors) or jointly (grey columns). This suggests that under all these conditions the two best reference genes are sufficient for effective data normalization.

Effective normalization of RT qPCR data requires the use of more than one reference gene [19]. For this purpose, GeNorm can compute a normalization factor *NF* based on geometric averaging of the expression levels of multiple reference genes. The software also provides a tool to determine the optimal number of reference genes to be employed for normalization. Starting with the *NF* obtained based on expression data for the two best reference genes with the lowest *M* values, new *NF* values are computed after the stepwise inclusion of additional reference genes in the order of the *M* value ranking. The optimal number of reference genes (n) is reached, when the inclusion of the next reference gene (n+1) fails to significantly affect the *NF* value, which is indicated by a pairwise variation value (*Vn/n+1*) below 0.15. Figure 4 shows that for the RT qPCR analysis of candidate reference gene expression based on all 10 *P. patens* RNA samples, GeNorm returns a *V2/3* value of only 0.104, suggesting that the two best reference genes (Ade PRT and 60S RIB; figure 2) are sufficient for effective normalization. *V2/3* values clearly below 0.15 were also obtained when candidate reference gene expression in different structures (*V2/3* = 0.124, figure 4), or in protonemata treated with hormones or hormone transport inhibitors (*V2/3* = 0.066, figure 4), was separately analyzed. Also under these conditions, effective normalization therefore requires only the two most stably expressed references genes (Ade PRT and 60S RIB, or Ade PRT and E3, respectively; figure 3).

### NormFinder Analysis of Reference Gene Expression Stability

The same data set used by GeNorm to generate the ranking shown in figure 2 was also analyzed by another software called NormFinder to determine candidate reference gene expression stability. NormFinder algorithms are not based on pairwise comparison of reference gene expression, but directly analyze expression variations within groups of candidate reference genes to assign an expression stability value to each of them [20]. NormFinder ranks the *P. patens* candidate reference genes analyzed in this study as shown in table 3. The same genes were ranked as the 7 most stably expressed reference genes both by GeNorm (figure 2, grey area) and NormFinder (table 3), although they are placed in a different order. Exactly these 7 genes were also identified by GeNorm as stably expressed suitable reference genes with *M* values below 0.5, when gene expression was separately analyzed in different *P. patens* structures and in protonemata treated with hormones or with hormone transport inhibitors (figure 3, grey area).

**Table 3:** NormFinder ranking of candidate reference gene expression stability.

N°	Gene name	Stability value
1	ST-P 2a	0,151
2	vH+PP	0,193
3	EF1α	0,264
4	Ade PRT	0,264
5	60S RIB	0,275
6	ACT	0,277
7	E2	0,284
8	TUA	0,581
9	E3	0,673
10	ARC34	0,898
11	SQS	1,287
12	SACPD	1,326

Expression stability values computed by NormFinder [11] are shown. Low stability values indicate stable gene expression. Genes are listed top to bottom in the order of decreasing expression stability.

## Discussion

A group of 7 stably expressed *P. patens* genes have been identified (Ade PRT, 60S RIB, E2, ST-P 2a, vH+PP, EF1α, ACT), which can be used for the normalization of data obtained by RT qPCR based gene expression analysis both in a range of gametophytic structures at different developmental stages and in protonemata treated with hormones or hormone transport inhibitors. The classification of these genes as suitable reference genes for RT qPCR analysis of gene expression in *P. patens* under a variety of conditions is supported by output generated by two different software packages, which are commonly employed to select the most stably expressed genes among groups of candidate reference genes, and which utilize distinct principles and algorithms for this purpose (GeNorm, NormFinder). Furthermore, GeNorm analysis suggests that the two most stably expressed references genes are sufficient for effective data normalization under all tested conditions. According to GeNorm output, these two genes were Ade PRT and E3 when gene expression was selectively investigated in protonemata treated with hormones or hormone transport inhibitors, or Ade PRT and 60S RIB when only gene expression in different structures was taken into account and when all samples were collectively analyzed.

Reference genes commonly used to date for RT qPCR based gene expression analysis in different plant model systems including *P. patens* were not among the most stably expressed reference genes identified in this study (ACT, EF1α), or were even found to be unsuitable for the normalization of RT qPCR data (TUA). This underscores the importance of identifying a specific set of stably expressed reference genes for each organism to be investigated by RT qPCR.

Although most of the 7 suitable *P. patens* reference genes identified in this study are moderately expressed, they cover a relatively large range of expression levels with a factor of 45x (2^5.5^) between the lowest and the highest expression level. Interestingly, this group of stably expressed reference genes appears to be composed not only of classical housekeeping genes, but also contains two genes coding for proteins with possible signaling functions (Ade PRT and ST-P 2a), which were actually ranked among the most stably expressed genes both by GeNorm (rank 1 and 4, respectively; figure 2) and by NormFinder (rank 4 and 1, respectively; table 3). This suggests that putative signaling functions of the proteins encoded by these genes may be primarily regulated at the post transcriptional level.

Gene expression levels were generally clearly more variable in different *P. patens* structures, than in protonemata treated with hormones or hormone transport inhibitors. This is exemplified by the E3 gene, which according to GeNorm analysis is the 2^nd^ most stably expressed gene when only protonemata treated with hormones or hormone transport inhibitors are analyzed, but was not included into the group of the 7 best reference genes identified by GeNorm and NormFinder because of its highly variable expression in different *P. patens* structures. Before RNA isolation, protonemata were grown on a different culture medium and for a shorter period of time than all other *P. patens* structures analyzed in this study (table 1). This was clearly not the only reason for the observed high variability of gene expression in these different structures, as GeNorm re-analysis of reference gene expression in all structures except for protonemata only resulted in a slightly reduced variability (data not shown).

As the study presented here was restricted to the analysis of 12 selected candidate genes, the list of suitable reference genes it produced is inevitably biased and non-exhaustive. Other *P. patens* genes may be equally or even more stably expressed under the conditions tested. However, as discussed above, the 7 identified suitable *P. patens* reference genes are more than sufficient for the effective normalization of RT qPCR data representing transcript levels in different *P. patens* gametophytic structures and in protonemata displaying altered hormone levels or transport. These references genes are stably expressed in structures as diverse as whole gametophytes, leafy shoots, rhizoids, protonemata grown on plain medium and protonemata displaying a range of striking hormone induced morphological alterations. They therefore appear likely to be stably expressed also under conditions other than those tested in this study, and to be suitable for a wider range of gene expression analyses. In any case, the stably expressed *P. patens* RT qPCR reference genes identified in this study are certain to enhance the quality of information collected concerning the control of gene expression in this important model organism.

## Methods

### Tissue Culture

All *P. patens* cultures were grown axenically in 10 cm plastic Petri dishes at 25°C under continuous white light with an intensity of 50 µmol m^−2^s^−1^. To initiate new cultures, small explants or homogenates prepared from protonemal pre-cultures grown for 7 days on solid BCDA medium (1 mM MgSO_4_, 1.85 mM KH_2_PO_4_, 10 mM KNO_3_, 45 µM FeSO_4_, 1 mM CaCl_2_, 0.22 µM CuSO_4_, 0.19 µM ZnSO_4_, 10 µM H_3_BO_4_, 0.10 µM Na_2_MoO_4_, 2 µM MnCl_2_, 0.23 µM CoCl_2_, 0.17 µM KI, 5 mM [NH_4_]_2_C_4_H_4_O_6_ and 0.7% agar) were sub-cultured on fresh plates. To grow whole gametophytes, small explants of protonemal pre-cultures (inocula) were transferred to solid BCD medium (BCDA medium lacking 5 mM [NH_4_]_2_C_4_H_4_O_6_) and cultured for 42 days. Protonemal cultures were grown for 7 days after spreading pre-cultures homogenized using a blender (TH-02; OMNI International, Kennesaw, GA, USA) on cellophane covered solid BCDA medium. Either IAA, ABA, 6-BAP, NPA or TIBA at a concentration of 5 µM, or NAA at a concentration of 1 µM, was added to the BCDA medium before the initiation of protonemal cultures to analyze effects of hormones or hormone transport inhibitors.

### RNA Extraction and cDNA Synthesis

Total RNA was extracted from plant material snap frozen and ground in liquid nitrogen using RNeasy Plant Mini Kits and DNase I treatment according to manufacturer’s instructions (Qiagen, Germany). Concentration, purity and integrity of isolated RNA samples was assessed and confirmed by determining OD_260_/OD_280_ ratios using a NanoVue Plus Spectrophotometer (GE Healthcare) as well as by 1% agarose gel electrophoresis. 500 ng of each RNA sample was reverse transcribed using iScript™ cDNA Synthesis Kits (Bio-Rad) according to the manufacturer’s instructions, and subsequently diluted 250 times in nuclease free water.

### DNA Extraction

Genomic DNA was extracted from 7 days old protonemal cultures snap frozen and ground in liquid nitrogen using the DNeasy Plant Mini Kit (Qiagen) following the manufacturer’s instructions. DNA concentrations were determined using a NanoVue Plus Spectrophotometer (GE Healthcare) and adjusted to 10 ng/µl with nuclease free water.

### Primer Design

Primers were designed with the help of Perlprimer software [53] to obtain melting temperatures (Tm) ranging from 59 to 61°C and fragment with sizes of 100 to 250 bp amplified from single exons. This ensured comparable results of fragment amplification from gDNA and cDNA templates.

### Real-time Quantitative PCR

RT qPCR was performed in a 96 well thermocycler (C1000 Touch™ Thermal Cycler, CFX96™ Real-Time System, Bio-Rad) using the iQ™ SYBR® Green Supermix (Bio-Rad). The thermocycler was programmed to run for 3 min at 95°C, followed by 40 cycles of 10 s at 95°C and 30 s at 60°C. Three identically treated replicas of each RNA sample were analyzed to account for biological variation, and each replica was run twice on the thermocycler to correct for technical variation. All RT qPCR data for the characterization of individual candidate reference genes were acquired simultaneously in single runs. Genomic DNA serially diluted to concentrations ranging from 36 to 46660 genome copies per well was used as a quantification standard and to test amplification efficiency (table 2). Specific amplification of single fragments of all reference genes was confirmed by recording a melting curve upon heating each PCR product from 60°C to 95°C (table 2) and by sequencing. Attempts to amplify non-coding regions of the *P. patens* genome were not successful using any of the analyzed RNA samples as a template, confirming that none of these samples was contaminated with genomic DNA.

### Computation of Gene Expression Stability and Number of Reference Genes Required for Normalization

Ct values indicating level of gene expression were determined by RT qPCR amplification of each candidate reference gene from all RNA samples. Mean Ct values and standard deviations computed based on the analysis of 3 biological replicates are listed in table S1. The range of all mean Ct values obtained for each candidate reference gene representing its level of expression in the different RNA samples (horizontal rows in table S1) is graphically displayed in figure 1. All mean Ct values were collectively analyzed by GeNorm (qbase^PLUS^ version 2.4, Biogazelle, Belgium) and by NormFinder (version 0.953, http://www.mdl.dk/publicationsnormfinder.htm) software following published procedures [19, 20; qbase^PLUS^ manual, Biogazelle, Belgium] to compute gene expression stability values (GeNorm, NormFinder), as well as pairwise variations of *NF* values (GeNorm), based on which numbers of reference genes required for effective normalization could be estimated.

## Supporting Information

Figure S1 Gametophytic *P. patens* structures analyzed for candidate reference gene expression. Seven day old *P. patens* protonemata cultured on BCDA medium covered with a cellophane disc (A, B). These protonemata were mainly composed of chloronemal cells (B), but also contained few cells with caulonemal character (arrow in B). Whole gametophytes grown for 42 days on BCD medium were composed of protonemata (largely hidden under gametophores) and gametophores (leafy shoots with attached rhizoids) (C; arrow: rhizoids). Isolated gametophores (D) were cut along the dotted lines to collect leafy shoots (above upper dotted lines) or rhizoids (below lower dotted line). Scale bars: 1 cm (A), 200 µm (B), 5 mm (C) and 3 mm (D).
(TIF)

Figure S2 Effects of hormones and hormone transport inhibitors on protonemata. Seven day old *P. patens* protonemata cultured on plain BCDA medium (A), or in the presence of hormones (B–E). (B) 5 µM 6-BAP promotes the development of numerous gametophore buds (arrows) [9], which are never observed at this developmental stage in non-treated cultures. (C) 5 µM ABA leads to the formation of short cells with a dense cytoplasm, which particularly at filament ends are also clearly wider than untreated cells [11]. (D) 5 µM IAA and (E) 1 µM NAA induce protonemata to prematurely develop caulonemal character, and effectively block lateral branching [7]. The auxin transport inhibitors NPA and TIBA at a concentration of 5 µM, at which they strongly affect the development of vascular plants [38], do not detectably alter the morphology of protonemata (not shown), but induce significant changes in the expression of a number of candidate reference genes (e.g. SQS, TUA) in these structures (F), as determined by normalization of the data shown in based on the two best reference genes using qbase^PLUS^. Scale bar: 500 µm; error bars: standard deviation; asterisks: statistically significant (t-test: P value <0.05) effects of hormone transport inhibitors on gene expression.
(TIF)

Table S1 Expression level of *P. patens* candidate reference genes as determined by RT qPCR.
(DOCX)
