# Supplementary figures and images for: Evaluation of Reference Genes for RT qPCR Analyses of Structure-Specific and Hormone Regulated Gene Expression in Physcomitrella patens Gametophytes
Source: PLoS One. 2013 Aug 9;8(8):e70998. doi: 10.1371/journal.pone.0070998 (PMC3739808; doi:10.1371/journal.pone.0070998)

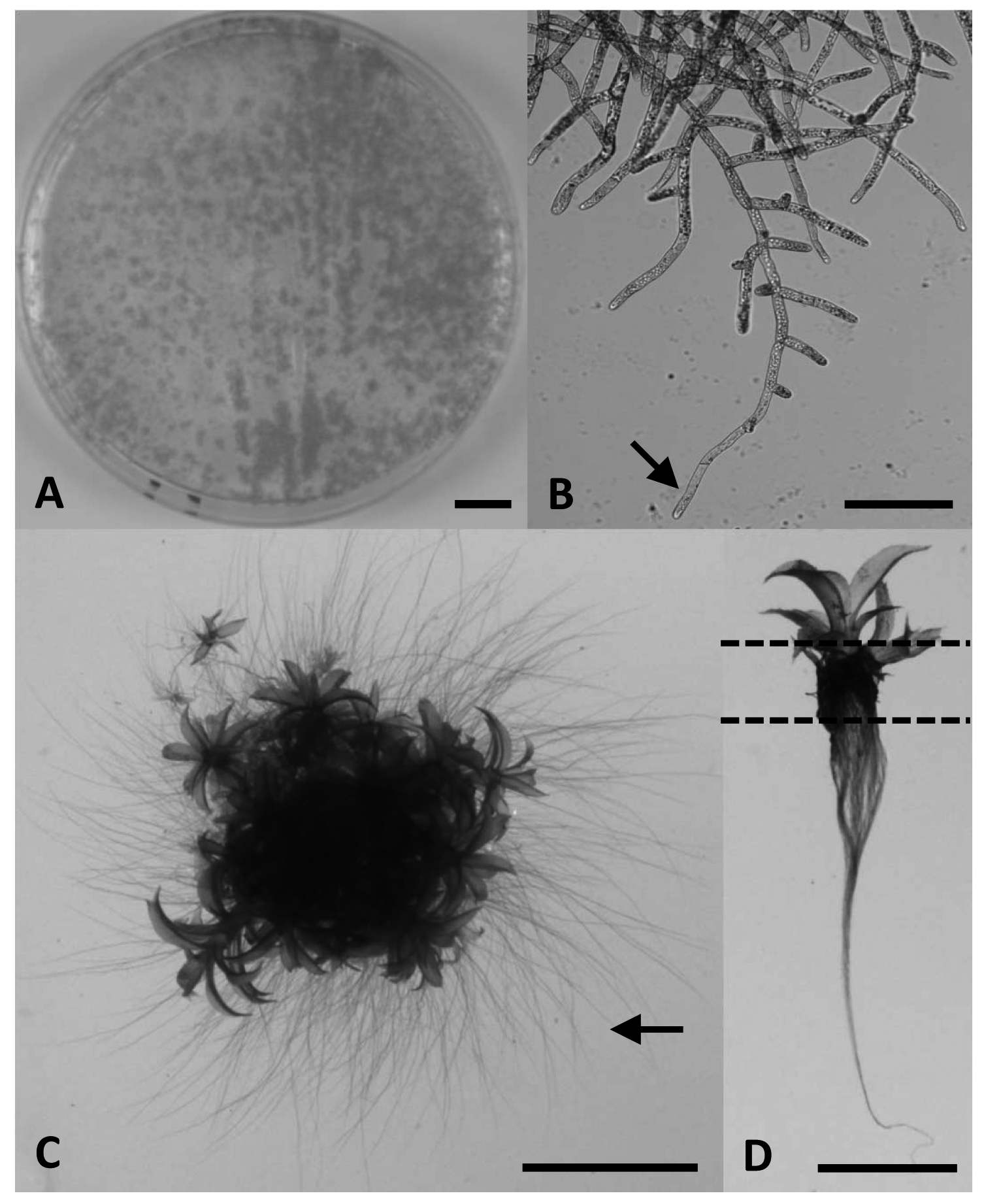

Supplement: Figure S1 — Gametophytic P. patens structures analyzed for candidate reference gene expression. Seven day old P. patens protonemata cultured on BCDA medium covered with a cellophane disc (A, B). These protonemata were mainly composed of chloronemal cells (B), but also contained few cells with caulonemal character (arrow in B). Whole gametophytes grown for 42 days on BCD medium were composed of protonemata (largely hidden under gametophores) and gametophores (leafy shoots with attached rhizoids) (C; arrow: rhizoids). Isolated gametophores (D) were cut along the dotted lines to collect leafy shoots (above upper dotted lines) or rhizoids (below lower dotted line). Scale bars: 1 cm (A), 200 µm (B), 5 mm (C) and 3 mm (D). (TIF) [file pone.0070998.s001.tif]

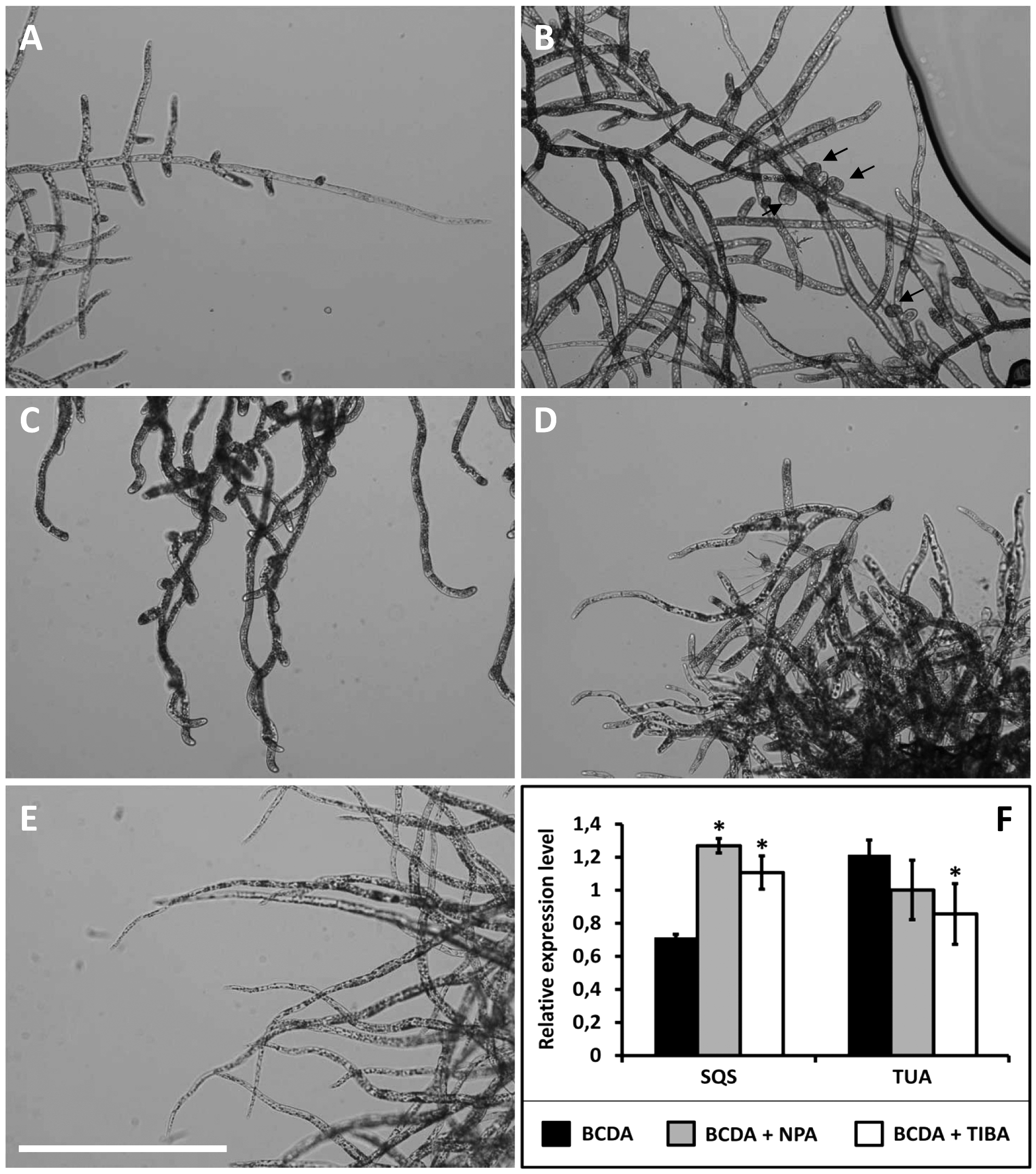

Supplement: Figure S2 — Effects of hormones and hormone transport inhibitors on protonemata. Seven day old P. patens protonemata cultured on plain BCDA medium (A), or in the presence of hormones (B–E). (B) 5 µM 6-BAP promotes the development of numerous gametophore buds (arrows) [9], which are never observed at this developmental stage in non-treated cultures. (C) 5 µM ABA leads to the formation of short cells with a dense cytoplasm, which particularly at filament ends are also clearly wider than untreated cells [11]. (D) 5 µM IAA and (E) 1 µM NAA induce protonemata to prematurely develop caulonemal character, and effectively block lateral branching [7]. The auxin transport inhibitors NPA and TIBA at a concentration of 5 µM, at which they strongly affect the development of vascular plants [38], do not detectably alter the morphology of protonemata (not shown), but induce significant changes in the expression of a number of candidate reference genes (e.g. SQS, TUA) in these structures (F), as determined by normalization of the data shown in based on the two best reference genes using qbasePLUS. Scale bar: 500 µm; error bars: standard deviation; asterisks: statistically significant (t-test: P value <0.05) effects of hormone transport inhibitors on gene expression. (TIF) [file pone.0070998.s002.tif]
