# Supplementary material for: Evaluation of Reference Genes for RT qPCR Analyses of Structure-Specific and Hormone Regulated Gene Expression in Physcomitrella patens Gametophytes
Source: PLoS One. 2013 Aug 9;8(8):e70998. doi: 10.1371/journal.pone.0070998 (PMC3739808; doi:10.1371/journal.pone.0070998)
Supplement: Table S1 — Expression level of P. patens candidate reference genes as determined by RT qPCR. (DOCX) [file pone.0070998.s003.docx]

**Supplemental table S1. Expression level of *P. patens* candidate reference genes as determined by RT qPCR.**

|  | protonemata | | protonemataIAA | | protonemata NAA | | protonemata NPA | | protonemata TIBA | | protonemata ABA | | protonemata6-BAP | | rhizoids | | leafy shoots | | whole plants | |
| --- | --- | --- | --- | --- | --- | --- | --- | --- | --- | --- | --- | --- | --- | --- | --- | --- | --- | --- | --- | --- |
| 60S RIB | 29,33 | ±0,14 | 29,45 | ±0,43 | 29,01 | ±0,21 | 28,91 | ±0,07 | 29,02 | ±0,13 | 28,86 | ±0,08 | 28,88 | ±0,16 | 29,12 | ±0,23 | 32,25 | ±1,09 | 31,36 | ±0,98 |
| ACT | 26,28 | ±0,29 | 27,47 | ±0,68 | 26,78 | ±0,16 | 25,95 | ±0,09 | 26,78 | ±0,10 | 25,86 | ±0,16 | 26,02 | ±0,16 | 26,79 | ±0,15 | 29,50 | ±0,68 | 28,57 | ±0,77 |
| Ade PRT | 25,60 | ±0,18 | 26,07 | ±0,31 | 25,73 | ±0,18 | 25,76 | ±0,29 | 25,91 | ±0,12 | 25,63 | ±0,31 | 25,52 | ±0,32 | 26,22 | ±0,72 | 28,52 | ±1,93 | 29,12 | ±0,24 |
| ARC 34 | 31,66 | ±0,37 | 31,87 | ±0,84 | 29,84 | ±0,27 | 31,01 | ±0,59 | 31,35 | ±0,63 | 30,64 | ±0,09 | 30,20 | ±0,12 | 29,91 | ±0,24 | 35,08 | ±2,34 | 32,96 | ±1,61 |
| E2 | 25,89 | ±0,06 | 26,32 | ±0,46 | 25,45 | ±0,29 | 25,58 | ±0,13 | 25,83 | ±0,10 | 26,02 | ±0,10 | 25,45 | ±0,19 | 24,99 | ±0,19 | 28,74 | ±1,94 | 27,84 | ±1,75 |
| E3 | 24,94 | ±0,05 | 25,33 | ±0,51 | 24,60 | ±0,29 | 25,08 | ±0,21 | 25,27 | ±0,12 | 24,89 | ±0,01 | 24,46 | ±0,15 | 24,17 | ±0,24 | 25,29 | ±0,29 | 24,58 | ±0,47 |
| EF1a | 24,78 | ±0,09 | 25,72 | ±0,60 | 25,32 | ±0,31 | 24,69 | ±0,22 | 24,99 | ±0,15 | 24,90 | ±0,09 | 24,70 | ±0,06 | 25,47 | ±0,14 | 27,34 | ±0,57 | 26,74 | ±0,72 |
| SACPD | 30,11 | ±0,11 | 29,82 | ±0,97 | 29,85 | ±0,21 | 29,72 | ±0,36 | 29,64 | ±0,58 | 25,10 | ±0,18 | 28,96 | ±0,05 | 28,76 | ±0,28 | 27,28 | ±1,05 | 28,65 | ±0,54 |
| SQS | 27,92 | ±0,11 | 28,71 | ±0,83 | 27,13 | ±0,35 | 27,17 | ±0,17 | 27,61 | ±0,25 | 27,97 | ±0,08 | 27,64 | ±0,16 | 28,02 | ±0,55 | 33,62 | ±2,06 | 33,41 | ±2,34 |
| ST-P 2a | 27,51 | ±0,26 | 27,77 | ±0,88 | 26,63 | ±0,15 | 26,93 | ±0,08 | 27,36 | ±0,11 | 27,30 | ±0,25 | 26,66 | ±0,07 | 26,58 | ±0,29 | 29,89 | ±1,15 | 28,92 | ±0,95 |
| TUA | 29,79 | ±0,17 | 30,55 | ±0,77 | 30,16 | ±0,25 | 30,16 | ±0,46 | 30,63 | ±0,19 | 29,01 | ±0,17 | 29,13 | ±0,14 | 28,79 | ±0,06 | 31,13 | ±0,81 | 29,83 | ±0,77 |
| vH+PP | 28,23 | ±0,28 | 28,82 | ±1,23 | 27,48 | ±0,17 | 27,69 | ±0,18 | 27,97 | ±0,12 | 27,90 | ±0,05 | 27,46 | ±0,16 | 27,38 | ±0,32 | 30,05 | ±1,54 | 29,31 | ±1,07 |

RT qPCR amplification of all candidate reference genes from different RNA samples: mean Ct value and standard deviation computed based on the analysis of 3 biological replicates.
